# Between Steps and Emotions: Folk Dance as a Promoter of Youth Well-Being

**DOI:** 10.3390/children13020211

**Published:** 2026-01-31

**Authors:** Karen Urra-López, Catalina Coronado-Reyno, Alda Reyno-Freundt

**Affiliations:** 1 Departamento de Educación Física Deportes y Recreación, Facultad de Artes y Educación Física, Campus Joaquín Cabezas García, Universidad Metropolitana de Ciencias de la Educación, Santiago 8320000, Chile; alda.reyno@umce.cl; 2 Facultad de Educación y Ciencias Sociales, Universidad Andrés Bello, Santiago 7591538, Chile; c.coronadoreyno@uandresbello.edu

**Keywords:** folk dance, psychological well-being, body expression, cultural identity, physical education

## Abstract

**Highlights:**

**What are the main findings?**

- School folk dance promotes the emotional well-being of children and adolescents, generating positive emotions, self-esteem, and psychological balance through movement.
- Students perceive folk dance not only as an artistic activity but also as a space for bodily and cultural expression that fosters identity, social cohesion, and self-confidence.

**What is the implication of the main finding?**

- Systematically incorporating folk dance into physical education can strengthen students’ mental health, motivation, and meaningful learning.
- Folk dance emerges as an integral pedagogical strategy that combines movement, emotion, and culture to promote youth well-being.

**Abstract:**

**Background/Objectives:** Folk dance represents an educational and cultural practice that is capable of promoting psychological well-being, social cohesion, and identity formation. However, few studies have integrated students’ voices regarding their lived experiences in these practices. This study aimed to analyze the perceptions of children and adolescents about their participation in school folk dances, exploring their impact on psychological well-being, self-confidence, and body awareness. **Methods:** A qualitative study with an exploratory and descriptive design was conducted with a purposive sample of 76 elementary and secondary school students who participated in the School Folk Dance Encounter “*Heartbeats of My Land*”, organized by the Metropolitan University of Educational Sciences (Chile). Semi-structured interviews were applied, and a thematic analysis was performed on 285 statements, organized into two dimensions: Psychological Well-being and Self-Confidence (**PWS**) and Body Awareness, Expression, and Communication (**CEC**). **Results:** The analysis revealed a predominance of the (**PWS**) dimension (85.3%), focused on positive emotions, self-confidence, and emotional regulation. Students’ testimonies highlighted dance as a means of release, self-esteem, and joy. To a lesser extent (14.7%), the (**CEC**) dimension reflected the perception of the body as a vehicle for communication and symbolic expression. **Conclusions:** Folk dance emerges as an integral pedagogical space that enhances emotional well-being, self-confidence, and cultural identity. Its systematic inclusion in Physical Education is proposed as a strategy to foster meaningful learning, mental health, and social cohesion.

## 1. Introduction

Folk dance can be defined as a traditional cultural expression transmitted across generations, serving to preserve the identity, values, and collective memory of a community [1,2]. These dance expressions take on diverse forms depending on historical, geographical, and social contexts, which explains why each country and even each region within the same territory develops its own styles, techniques, rhythms, and meanings [3]. In this sense, folk dance functions both as an artistic practice and as a vehicle for cultural transmission that shapes belonging, tradition, and social continuity [3].

The implementation of folk dance holds great importance due to its multiple characteristics and benefits that foster the holistic development of individuals [4]. This artistic and cultural practice generates a multidimensional positive impact, encompassing the emotional, physical, intellectual, spiritual, and relational spheres [5,6,7]. It contributes to the individual’s overall health and functions beneficially across all ages, genders, and socio-cultural and socioeconomic contexts [4].

Understanding students’ perceptions of folklore—the meaning they assign to it, the value they attribute to it, and the conditions that either facilitate or hinder their participation—is key to aligning curricular intentions with actual experience. The *student voice* literature supports positioning students’ perspectives as a direct input for improving teaching practices and co-constructing curricula [8,9]. Meanwhile, expectancy–value motivational frameworks provide interpretive lenses to understand how competence beliefs and perceived value are configured, thereby conditioning engagement with the practice [10,11].

This *student voice* perspective gains particular relevance in the context of folklore for reasons involving engagement, holistic development, and cultural preservation [4,12]. When educators understand students’ motivations and interests—whether it is their love for dance, passion for traditional culture, desire for physical exercise, or the release of stress—they can adapt activities to foster active participation, transforming folk dances into rich and meaningful learning experiences [12,13,14,15]. This understanding allows for concrete pedagogical guidance in the design, sequencing, and assessment of folklore within Physical Education. In this regard, it is important to understand two areas in which folklore has a significant impact.

### 1.1. Personal Well-Being

Folk dance provides both psychological and physical relaxation, serving as an effective means to enhance self-perception, strengthen self-confidence, reduce anxiety and depression, and develop social and emotional skills [7,12,16,17].

Recent studies by Jochum et al. [12] suggest that folk dance has the potential to foster psychological well-being by promoting greater sociability and reducing social isolation. Regarding self-confidence and self-esteem, Urra et al. [6] describe dance as a catalyst for improving self-esteem and interpersonal relationships in school contexts, as it contributes to personal validation. Furthermore, comparative studies between young dancers and other groups indicate that, although dancers do not necessarily surpass athletes in perceived well-being, they tend to value the emotional benefits of the activity more intensely and exhibit a stronger connection with their bodies and self-image [18].

### 1.2. Coordinative and Physical Abilities

Folk dance stands out for its capacity to enhance overall physical fitness [5,6]. In the specific case of children, this discipline significantly promotes the development of muscle tone, laterality, balance, and body awareness [19].

A recent systematic review concluded that traditional dance significantly improves body coordination, balance, body perception, and spatial awareness in children [3]. Likewise, specific studies have reported that structured dance programs—including folk genres—enhance motor coordination and balance in preschool-aged children [20]. These findings support the view that dance is not only a form of cultural expression but also an effective tool for strengthening the foundations of psychomotor development in early childhood.

Regarding dance as a psychomotor strategy, Soares et al. [19] note that Physical Education classes constitute a pedagogical tool that enables the structuring of essential psychomotor functions for children’s holistic formation. This process contributes to greater mastery of general motor coordination, balance, temporal and spatial awareness, and improved body consciousness. Even in online formats during the COVID-19 pandemic, Urra et al. [6] reported benefits for students, including the stimulation of overall motor skill development, enhancement of laterality, spatial orientation, and sense of rhythm.

Some studies have revealed that students often show limited understanding and interest in folklore, which may affect the effectiveness of teaching and learning processes [7]. From a curricular standpoint, the sustained integration of folklore faces operational gaps: there is a shortage of specific didactics and reduced presence or evaluative requirements regarding folk dance within Physical Education. This makes it difficult to establish robust didactic criteria and sequential progression [14]. Consequently, incorporating folklore beyond commemorative events faces persistent barriers. Teachers report the absence of clear evaluation guidelines and structured programs that ensure longitudinal progression, resulting in isolated and unsustainable practices [21,22].

Based on the foregoing, several questions arise: Do students’ narratives validate the benefits that contemporary scientific literature attributes to the practice of folk dance? Are there elements in these narratives that contrast with, complement, or expand upon established academic findings? Which aspects of the student experience require deeper research exploration? This study therefore seeks to determine whether the narratives of children and adolescents who systematically participate in school folk dance are consistent with the findings of recent research, thus establishing a dialog between the lived experiences of participants and the scientific knowledge produced about this educational and cultural practice.

## 2. Materials and Methods

A semi-structured interview composed of four open-ended questions was administered. Although the questions addressed different aspects of folklore, they all converged toward a single central objective: accessing the perceptions, emotions, and meanings that students attribute to the practice of folk dance.

*The questions were as follows:*

- “What are the reasons that led you to choose to dance folk dances?”
- “What do you think is the relationship between folk dances and a country’s history?”
- “How important is it to you whether a Chilean folk dance is or is not related to politics?”
- “What do you feel when you dance folk dances?”

These four questions were conceptually organized into four analytical axes, which guided the subsequent construction of categories:

- Emotions and sensations experienced while dancing folklore (directly derived from Question 4, the core of the study).
- Personal perception of participation in the activity (related to Question 1).
- Value and meaning attributed to folk dance (articulated through Questions 1, 2, and 3).
- Relevance of folklore in school and everyday life, including cultural and identity aspects (Question 2 and part of Question 3).

The analytical emphasis of the study was primarily placed on the emotions and subjective experiences expressed in Question 4, as it directly relates to the research question. However, the other three questions complemented and enriched the understanding of the phenomenon, allowing for the triangulation of meanings and deeper development of the emerging categories.

This format enabled the collection of spontaneous and in-depth narratives, keeping the focus on the student’s subjective experience without steering their responses toward predefined content.

*Methodological Rigor (Revised and Strengthened). Pilot Test/Validation.*

No formal pilot test was conducted because the questions used are open-ended items commonly employed in qualitative research on student perceptions, and they have proven effective in exploring cultural and emotional meanings in school contexts.

*Validation was ensured through:*

- Content review by the research team.
- Discussion of the semantic relevance of each question.
- Direct alignment with the research question.
- Data Saturation.

*Saturation was reached when narratives began to repeat clear thematic patterns related to:*

- Emotional well-being.
- Cultural meaning of dances.
- Subjective experiences.
- Bodily expression.

When no new relevant elements emerged, the interview volume was considered sufficient to answer the research question.

*Reflexivity.*

The research team held consensus meetings during the analysis, where they discussed:

- Potential interpretive biases.
- Alignment between emerging categories and original data.
- Coherence between the applied questions and the study objectives.

This process ensured a transparent interpretation, preventing personal perspectives from influencing the results [23].

### 2.1. Presentation of the Dimensions and Categories

#### 2.1.1. Dimension 1: Psychological Well-Being and Self-Confidence (**PWS**)

This dimension refers to the subjective state experienced by students during the practice of folk dances, where the regulation of emotions, the experience of positive affects, and self-appreciation converge. It encompasses the ability to maintain emotional balance (reduction in anxiety, stress management, and release of tension), the experience of pleasant affective states (joy, interest, happiness, and well-being), and a positive self-perception that emerges both from the interpretation of one’s own qualities and achievements and from social validation (applause, praise, and external recognition). Collectively, these elements contribute to strengthening psychological well-being, personal confidence, and resilience.

Category: Emotional Balance (**EBA**)

This category identifies and quantifies statements reflecting reductions in anxiety, stress management, and emotional release. It denotes inner harmony and a sense of enjoyment.

*Example: “It clears my mind from everything bad that has happened during the week. Dancing clears me from it all.”*

Category: Positive Affective States (**PAS**)

This category captures and quantifies manifestations of happiness, interest, joy, well-being, and heightened emotional states experienced during dance practice.

*Example: “Dancing makes me feel like a spark inside… It fills me. That spark starts to generate warmth, and what it really produces is happiness.”*

Category: Self-appreciation (**SAA**)

This category identifies and quantifies the perception, judgment, and self-recognition that a person has of themselves, based on the interpretation of their qualities, capacities, achievements, and limitations, as well as the social feedback and validation received.

*Example: “I feel really beautiful when I dance folklore.”*

#### 2.1.2. Dimension 2: Body Awareness, Expression, and Communication (**CEC**)

This dimension refers to the development of awareness of one’s own body and that of others. It involves spatial utilization and the development of rhythmic sense, as well as experiences of transcendence and emotional release. It reflects the ability to express and communicate through the body.

Category: Body and Movement (**BMO**)

This category identifies and quantifies statements that reflect awareness of one’s own body in actions related to physical exercise, motor practice, and body image.

*Example: “Because it’s exercise and because I’ve always liked it—for obvious reasons.”*

Category: Body Communication and Expression (**BCE**)

This category identifies and quantifies statements that express the sensation of communicating through the body, conveying feelings and emotions.

*Example: “I believe that dancing expresses much more than words could ever say.”*

## 3. Results

The presentation of results is based on the responses provided by 76 students who participated in the *School Folk Dance Festival* “Heartbeats of My Land” The analysis was organized into two main dimensions and their corresponding categories (Table 1).

### 3.1. Dimension: Psychological Well-Being and Self-Confidence (**PWS**)

The information obtained for the Psychological Well-being and Self-confidence (**PWS**) dimension is presented in Table 2, grouping the data referring to the frequency of statements associated with Emotional Balance, Positive Affective States, and Self-Appreciation.

### 3.2. Dimension: Body Awareness, Expression, and Communication (**CEC**)

The information obtained for the Body Awareness, Expression, and Communication (BAEC) dimension is presented in Table 3, which groups the data referring to the frequency of statements associated with Body and Movement and Body Communication and Expression.

## 4. Discussion

### 4.1. Dimensions

In this study, a total of 285 statements were identified and grouped into two major analytical dimensions: Psychological Well-being and Self-confidence **(PWS)** and Body Awareness, Expression, and Communication **(BCE)**. The results revealed a clear predominance of the former, which concentrated 85.3% of the statements, while the latter accounted for 14.7%.

This contrast shows that students more frequently verbalize aspects related to psychological well-being and self-confidence, highlighting the importance of the affective dimension in the dance experience [24]. However, the lower percentage attributed to the bodily–expressive dimension should not be interpreted as irrelevant; rather, it illustrates that the psycho-emotional component is more easily articulated verbally, whereas experiences linked to bodily awareness and communication through movement tend to be more implicit, less verbalized, and often more difficult to describe [25].

**Figure 1:**
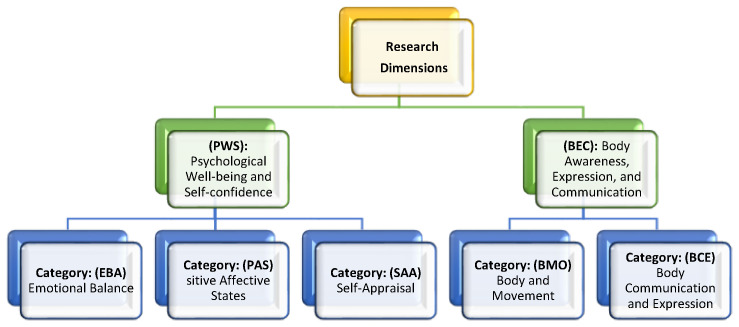
Dimensions and categories of study.

Despite its smaller quantitative weight, the (**BEC)** dimension plays a significant role in the overall configuration of the experience, as it positions the body as a vehicle for expression, communication, and motor perception—complementing the dimension of psychological well-being. In this sense, the coexistence of both dimensions in students’ narratives reflects the complex and multifaceted nature of folk dance, which not only fosters positive emotional states and strengthens self-confidence but also activates processes of bodily awareness, cultural identity, and symbolic communication (Figure 1).

The most significant finding of this research suggests that traditional dances transcend mere physical or expressive activity, becoming primarily a tool for emotional regulation and the construction of personal well-being. This result aligns with the postulates of Talpă [4] who emphasizes the role of dance movement as a mediator of psychological balance.

*Example: “Honestly, I feel really happy. I’ve always loved it since I was little, so I always feel happy, and I think I’ll always be happy dancing this.”*

The relevance of these findings is closely related to the foundations of dance therapy and positive psychology, disciplines that conceptualize structured body movement as a natural mechanism of emotional self-regulation. Buyrukoğlu [16] supports this theoretical–practical connection, emphasizing that dance significantly contributes to psychological outcomes related to mental health and overall well-being. In the specific context of folk dance [6], has documented its function as a promoter of cultural expression that enhances socio-emotional stimulation, generating a positive impact on both cognitive and emotional aspects of human development.

*Example: “I feel good; it makes me forget about everything—all the problems I go through. It’s like a way to de-stress. And especially with my group, when I feel really down, I come here, and they always make me smile. So, it’s awesome.”*

From a holistic perspective, dance conceived as a motor project transcends the mechanical analysis of steps and movements. Instead, it aims to give meaning to the cognitive and expressive levels of the individual, facilitating introspection and interpersonal connection through sensory exploration. This process allows for deeper contact with personal feelings and emotions, becoming a valuable instrument for self-knowledge and self-concept development, as well as an effective bridge for social *connection that enriches participants’ cognitive and emotional repertoire [26].*

*Example: “Emotion, happiness, love too—freedom of expression. Because I feel that through dance, I can express myself maybe not by talking, but by dancing.”*

The principles of positive psychology find in dance a privileged medium to foster self-appreciation, assertive communication, and mutual respect among students, contributing significantly to overall mood improvement. Kurutz et al. [27] observed that feeling actively involved in the dance learning process leads to substantial increases in self-esteem, while systematic practice fosters confidence and self-assurance, particularly among children and adolescents.

*Example: “I feel really good, I’m beautiful, I see something I like, and it makes me happy.”*

The concept of “experiential enjoyment” that emerges in the practice of dance transcends bodily movement and becomes a space for emotional release and subjective well-being. This experience confirms what Rannau-Garrido and Contreras-Olivares [14] pointed out: the hedonic component of dance fosters self-esteem and self-concept in children and adolescents, establishing it as both a pedagogical and therapeutic resource. Likewise, Koch et al. [28] demonstrated that dance—by integrating emotion, cognition, and corporeality—promotes emotional self-regulation, reduces stress, and improves mental health. Along these lines, Koch et al. [28] highlighted that a positive self-concept is strengthened when students engage in activities that generate personal satisfaction and reinforce identity. Therefore, experiential enjoyment is not merely an artistic goal but a mechanism of integral well-being with emotional and social impact.

*Example: “Happiness, and maybe a sense of release from the burdens one carries in school life. Peace—it’s like a way to relax.”*

Finally, contemporary neuroscientific evidence reinforces these findings, demonstrating that the stimulation derived from bodily expression and dance is associated with functional and structural changes at the brain level, confirming its fundamental role in psychological well-being. Soares et al. [16] documented that the expression of feelings and the structuring of psychomotor functions constitute integral formative possibilities offered by dance as a pedagogical and therapeutic strategy.

*Example: “Liberation. I feel like I can free myself and express what I feel through movement and dance in general joy.”*

### 4.2. Categories Corresponding to the “Psychological Well-Being and Self-Confidence (PWS)” Dimension: Positive Affective States (PAS), Emotional Balance (EBA), and Self-Appreciation (**SAA**)

The Psychological Well-being and Self-confidence (**PWS**) dimension is defined as the subjective state that emerges in the practice of folk dance through the integration of emotional regulation (reduction in anxiety, stress management, and tension relief), the experience of positive affects (joy, interest, well-being), and self-appreciation (self-esteem, recognition of achievements, and social validation).

Accordingly, the study results show that out of 243 statements associated with this dimension (85.3% of the total 285), the category Positive Affective States (**PAS**) accounts for 81.9%. This predominance highlights that the dance experience is characterized by intense and recurring pleasant emotions: *“I feel good; it makes me forget about all the bad things.”* This marked concentration in the positive emotional spectrum positions folk dance as a consistent and effective generator of pleasant affective experiences, a finding consistent with contemporary research by Rannau-Garrido and Contreras-Olivares [14], who have documented similar patterns in contexts of traditional bodily expression.

Similarly, recent studies indicate that dance practice promotes positive mood states and mitigates negative effects across diverse populations [29].

The Emotional Balance (**EBA**) category comprises 10.3% of the statements, representing expressions of relief, inner harmony, and stress regulation: *“It clears my mind of everything that’s happened... dancing clears me of it all.”* Although quantitatively smaller, its role is essential as an emotional buffer and facilitator of internal stability.

Numerous studies support that dance can reduce anxiety levels and foster adaptive mechanisms of emotional regulation [29].

The Self-Appreciation (**SAA**) category, which represents 7.9% of statements, includes expressions of competence, positive self-image, and external validation: *“Oh, I feel happy, and I like it when people applaud me or say, ‘Oh, you did great.’ That’s it.”* Such manifestations align with Bandura’s (postulates on verbal persuasion as a source of self-efficacy) [30] and with contemporary self-concept theories emphasizing the importance of social feedback in building self-esteem [31].

The prevalence of these positive affective states suggests that folklore transcends its purely choreographic dimension, functioning as an effective mechanism of emotional regulation. In this sense, the results align with [17], who argues that the externalization of emotions, feelings, and ideas through bodily movement significantly enhances the perception, understanding, and reflection of one’s own emotional state. This theoretical perspective becomes particularly relevant considering that folk dance provides a socially validated channel for the conscious elaboration of affective experiences.

The intensity and consistency of positive emotional states observed in this study suggest that folk dance acts as a catalyst for emotional well-being beyond the immediate pleasure associated with physical activity. This interpretation is supported by Conesa [32], who evidenced that folk dance functions as a multidimensional tool integrating physical, cultural, and emotional components to generate a holistic impact on participants’ well-being.

The data indicate that this predominance of positive affective states is not random but rather responds to intrinsic characteristics of folk dance as a cultural practice. Its ritual structure, communal dimension, and connection with identity elements converge to create an environment conducive to the emergence of positive emotions an affective ecosystem that transcends the immediate context of dance practice.

### 4.3. Categories Corresponding to the “Body Awareness, Expression, and Communication (BCE)” Dimension: Body and Movement (BMO) and Bodily Communication and Expression (**BCE**)

The **(BCE**) dimension refers to the development of awareness of one’s own body and that of others, spatial and rhythmic use, as well as the ability to transcend and release emotions through movement. It implies that students express and communicate with their bodies, integrating sensory experience, creativity, and symbolic projection.

The predominance of the Communication and Expression (**BCE**) category (64.3%) over the Body and Movement (**BMO**) category (35.7%) within the bodily dimension suggests that students value the communicative capacity of movement more than pure sensory experience: *“Emotion, lots of emotion. Love—all emotions together.”* This indicates that the value of movement lies in its capacity to transmit and externalize emotions. According to Laban [33], dance cannot be understood without its expressive dimension, as every gesture carries meaning and constitutes a form of symbolic language. Complementarily, Torrents and Castañer [34] propose that bodily expression in educational contexts plays a central role in holistic development, fostering identity, creativity, and social interaction. Thus, the apparent “underestimation” of bodily perception responds to students’ preference for experiencing movement as communication rather than as isolated sensory perception.

This finding may reflect an intuitive understanding of dance as a social language, where the capacity to communicate transcends individual motor experience. In the context of folklore, this is particularly meaningful since such dances are inherently communicative and narrative.

The identity dimension underlying the practice of folk dance stands out, linking it to personal satisfaction and the desire to belong to a cultural tradition. Cuervo-Zapata and González-Palacio [26] affirm that community artistic practices strengthen self-concept construction and consolidate a sense of cultural belonging. Similarly, Urrea-Giraldo and Hernández-Carvajal [35] show that traditional dances act as intergenerational bridges that preserve and renew the cultural identity of communities.

The (**BMO)** category, representing 35.7% of statements, centers on awareness of one’s body in action, particularly regarding motor practice, physical exercise, and body image perception. Unlike the CEX category—focused on symbolic and relational gestures (**BMO**) emphasizes movement itself as a physical and self-perceptive experience.

*Example: “Because of the exercise, and because I’ve always liked it—for obvious reasons.”*

Here, the emphasis lies on the motor dimension and the continuity of practice as a habit, reflecting that folk dance is also conceived as a physical activity with benefits associated with body movement. This result shows that, although minority compared to expressive aspects (64.3%), the (**BMO**) category offers a key perspective: folk dance is lived not only as a cultural language but also as a conscious bodily exercise. Recent studies underline that dance, by involving rhythmic motor activity, enhances physical condition, coordination, and body awareness, contributing to students’ holistic development [36].

The results suggest that the practice of folk dance articulates two fundamental axes: on the one hand, emotional and psychological well-being, which emerges with greater discursive strength; and on the other, embodied–expressive experience, which—although less frequently mentioned—constitutes an essential component of the dance experience as an educational, cultural, and social practice. Thus, folk dance, beyond its artistic and cultural function, is consolidated as a privileged space for psychological well-being and holistic formation. The marked predominance of the Psychological Well-being and Self-confidence dimension (85.3%) over Body Awareness, Expression, and Communication (14.7%) shows that students experience dance primarily as a practice that supports emotional regulation, self-esteem, and positive self-perception. This indicates that folklore is not limited to cultural preservation but also operates as a pedagogical and psychosocial tool with broad reach [37].

Although it might seem obvious that those who participate in dance workshops do so out of personal interest and enjoyment, this study shows that such motivation goes beyond mere pleasure. Voluntary participation in extracurricular spaces strengthens high levels of intrinsic engagement and sense of belonging, shaping meaningful learning environments in which emotional, cultural, and social development converge. In line with this, prior research has shown that organized participation in artistic activities strengthens identity, self-confidence, and the construction of self-concept in youth [26,37].

The concentration of statements on positive affective states confirms that folk dance functions as an emotional catalyst that generates pleasure, satisfaction, and resilience. This evidence is consistent with Koch et al. [28], who argue that dance integrates emotion, cognition, and corporeality, contributing to mental health and socioemotional well-being. Consequently, folklore acquires renewed educational value: it not only preserves traditions but also drives processes of subjective well-being and identity formation.

The findings also reinforce the importance of considering folk dance as a strategic resource for the pedagogy of well-being. Educational neuroscience has shown that positive emotional states not only foster intrinsic motivation but also enhance attention, memory, and creativity, optimizing learning processes [38,39]. In this sense, folk dance can be understood as an educational practice that activates emotional and cognitive circuits capable of strengthening students’ holistic well-being.

Although the embodied–expressive dimension was less verbalized, its contributions are not minor. Testimonies related to motor awareness and bodily exercise show that dance is also perceived as conscious physical activity, with benefits for coordination, physical fitness, and body perception [36]. Thus, the bodily and expressive components constitute the foundation that sustains and projects the emotional benefits.

Taken together, the results open new avenues for research and educational practice. Future work should examine the mechanisms that explain the predominance of psychological well-being and design pedagogical strategies that intentionally integrate the emotional, motor, and cultural benefits of folk dance. This would make it possible to harness its potential not only as a cultural practice but also as a comprehensive educational intervention with impact on mental health, social cohesion, and youth identity.

## 5. Conclusions

The results of this study show that the practice of folk dance constitutes a privileged space for students’ emotional well-being and subjective development, particularly highlighting the emergence of positive affective states and the perception of self-appreciation. Although the bodily–expressive dimension is verbalized less frequently, it remains an essential component that sustains the emotional and communicative experience of dance.

The educational relevance of these findings lies in the fact that folk dance—already incorporated into the Physical Education curriculum in Chile—not only preserves cultural practices but also fosters learning environments that promote emotional regulation, a sense of belonging, and identity integration among adolescents.

This study presents limitations that are inherent to its qualitative design, such as intentional sampling, the self-selection of students who are motivated by dance, and the festive context of the event—factors that may enhance the expression of positive emotions. Nevertheless, these conditions also make it possible to understand the phenomenon within its natural context and from the voices of the participants themselves.

It is recommended that future research explore the longitudinal nature of these effects, compare experiences across different styles of school-based dance, or incorporate diachronic analyses that allow for examining how emotional well-being associated with folk dance practice evolves over time.

## Figures and Tables

**Table 1:** Frequency of Statements by Dimension.

Dimensions	Frequency	Percentage of Occurrence (n = 285)
Psychological Well-being and Self-confidence (**PWS**)	243	85.30%
Physical Exercise, Expression, and Body Communication (**CEC**)	42	14.70%

**Table 2:** Frequency of Statements Related to the Categories of the Psychological Well-being and Self-confidence (**PWS**) Dimension.

Psychological Well-Being and Self-Confidence (PWS)	Frequency	Percentage of Occurrence (n = 243)
Category: Emotional Balance (**EBA)**	25	10.3%
Category: Positive Affective States (P**AS**)	199	81.9%
Category: Self-Appreciation (**SAA**)	19	7.9%

**Table 3:** Frequency of Statements Related to the Categories of the Body Awareness, Expression, and Communication (**CEC)** Dimension.

Body Awareness, Expression, and Communication (CEC)	Frequency	Percentage of Occurrence (n = 42)
Category: Body and Movement (**BMO)**	15	35.7%
Category: Body Communication and Expression (**BCE**)	27	64.3%

## Data Availability

The datasets generated and analyzed during the current study are not publicly available to protect the privacy of the participating children but may be provided by the authors upon reasonable request and with appropriate ethical approval.

## Abbreviations

The following abbreviations are used in this manuscript:

PWS	Psychological Well-being and Self-confidence
EBA	Emotional Balance
PAS	Positive Affective States
SAA	Self-appreciation
CEC	Body Awareness, Expression, and Communication
BMO	Body and Movement
BCE	Body Communication and Expression
